# Perceived barriers and facilitators of physical activity in adults living in activity-friendly urban environments: A qualitative study in Sri Lanka

**DOI:** 10.1371/journal.pone.0268817

**Published:** 2022-06-02

**Authors:** Madhawa Perera, Carukshi Arambepola, Fiona Gillison, Oliver Peacock, Dylan Thompson

**Affiliations:** 1 Department of Gynaecology and Obstetrics, Faculty of Medicine and Allied Sciences, Rajarata University of Sri Lanka, Anuradhapura, Sri Lanka; 2 Department of Community Medicine, Faculty of Medicine, University of Colombo, Colombo, Sri Lanka; 3 Department for Health, University of Bath, Bath, United Kingdom; National Research Centre of Egypt, EGYPT

## Abstract

**Background:**

Despite reforming health policies to create more enabling environments, insufficient physical activity in Sri Lanka remains a major public health issue. Socio-culture specific determinants underlying the physical activity of adults living in such environments need to be identified. The aim of this study was to explore the barriers and facilitators for physical activity, as perceived by adult urban dwellers in activity-friendly environments in Colombo District, Sri Lanka.

**Methods:**

A qualitative study using in-depth interviews was conducted among adults aged 20–60 years living in an urban area which has been recently re-designed for recreational and rejuvenating purposes in Sri Lanka. Recruitment targeted varying socio-economic status and risk of non-communicable diseases; and was continued until the data saturation point was reached. Interviews were conducted in homes, primary healthcare units and fitness centres, and were transcribed verbatim and analysed using framework analysis.

**Results:**

A total of 31 eligible and consenting adults were interviewed. Of the reported barriers to physical activity, lack of time was very common. Other frequently reported barriers included unpleasant experiences following exercise and misconceptions about exercise, whereas physical environmental factors, weather and road safety were reported less frequently. All participants reported at least one facilitator for engaging in exercise. Expectations of preventing diseases, improving health, physical fitness, psycho-social wellbeing, optimising body functions and increasing lifespan were frequently cited as reasons to be active, while social factors such as positive attitudes of family members and the influence of peers were found to be motivating.

**Conclusions:**

The study showed that while participants valued the health benefits of physical activity and refurbished activity-friendly urban environments, these were not sufficient to support them to overcome key perceived barriers to being physically active.

## Introduction

Physical inactivity is a major risk factor for chronic non-communicable diseases (NCD) [1]. It is identified as the fourth most prominent threat for more than 3 million deaths each year [2] and 13.4 million disability-adjusted life-years worldwide [3]. Such losses are difficult to be borne by developing countries especially in South Asia, where the prevalence of physical inactivity is relatively high (33% compared to 16–26% of other lower-middle-income countries) [4]. Against this backdrop, the promotion of physical activity (PA) represents a low-cost and effective strategy for preventing the onset and reducing the severity of chronic NCDs. To achieve these benefits, guidelines have been established to identify and educate people about optimal PA levels [5]. However, translating these recommendations into action has been challenging for healthcare providers and promoters all over the world [6].

Physical activity is determined by multiple factors that may be internal to the individual or external in nature and related to where individuals live and work [7]. It is shown that awareness on the potential benefits of PA encourages people to initiate exercise [8], however, an active lifestyle is challenging to sustain over the long-term and impacted by barriers that outweigh perceived benefits, or by benefits that become less apparent over time [9]. Thus, facilitators as well as barriers need to be well-recognised in programmes that promote PA [10].

Previous studies have identified a number of barriers to PA, of which factors related to urbanisation are more apparent in cities, where changes to the built environment, overcrowding, higher economic pursuits, sedentary occupation and safety issues contribute substantially towards a sedentary lifestyle [11, 12]. In response, many countries have given priority to transform such settings into places more conducive to being active [13]. Establishing walking paths, designated areas for exercise, public playgrounds and safe neighbourhoods are some of these initiatives that have shown to encourage people to lead active lives [14].

Sri Lanka, a country in South Asia has introduced PA-friendly urban planning improvements in its major cities since 2012 [15, 16]. Despite the modifications, physical inactivity has increased from 15.6% in 2003 to 28.9% by 2016 [4, 17], reflecting a rapidly expanding public health problem among urban-dwellers in Sri Lanka [17, 18]. This suggests that built environmental features alone may not orient health behaviours of individuals, thus highlighting the importance of understanding what drives people to choose the opportunities created within healthy cities. In addition, the barriers specific to South Asia, such as the traditional gender role of South Asian women in which greater responsibility is borne by them on child care, household chores and supporting extended family members, and other socio-cultural attitudes on PA [17, 19, 20] need to be identified and targeted to complement the prevailing environmental modifications.

Although barriers for PA have been well-studied among patients with chronic NCDs in South Asia [21, 22], less information is available on barriers and facilitators in apparently healthy adults and those at increased risk of chronic NCDs. Thus, the aim of this study was to explore the barriers and facilitators of PA among such urban dwellers in Colombo District of Sri Lanka.

## Materials and methods

### Design

This study is reported based on the guidelines provided in the consolidated criteria for reporting qualitative research (COREQ) [23]. This qualitative study was part of a mixed-methods pilot to assess the acceptability of technology-enabled personalized feedback on PA [24].

Qualitative data were collected from September to December in 2017 through in-depth interviews among adults living in a geographically defined administrative area within Sri Jayewardenepura Kotte, the Capital of Sri Lanka. This area comprises a population of varying socio-cultural and economic backgrounds; and recently introduced recreational areas, wetland parks, play areas, green walk-trails and urban bio-diversity refuges revitalizing the marshy land for the purpose of urban beautification, environmental improvement and eco-tourism, while providing the city dwellers an opportunity to relax, exercise and rejuvenate within the designated convivial places [15, 16].

### Participants

The inclusion criteria were for adults aged 20–60 years who had been living in the area for a minimum period of one year. Those diagnosed with heart disease or stroke (confirmed with documental evidence) or having physical disabilities (e.g., limb deformities, blindness), contra-indications for PA (e.g., medical reasons), mental disorders or acute illness, and pregnant women were excluded from the study.

The sample size required for the study was determined by the point at which no more new information was generated during the interviews (i.e., data saturation) [25]. To ensure the data saturation point was reached, three more participants were interviewed without new ideas emerging, before recruitment was stopped [25].

### Procedure

Ethical approval for the study was granted by Ethics Review Committee of the Faculty of Medicine, University of Colombo, Sri Lanka (EC-17-116).

The sampling was done purposively with the assistance of area public health midwives, during which a list of adults was pre-identified and used to approach the eligible persons until the study reached its data saturation point. An attempt was made to reflect as much as possible the variation of the target population by socio-economic status (SES), by recruiting participants from different neighbourhoods typically classified by the local community as ‘affluent’, ‘middle-income’ and ‘low-income’. Further, to reflect their variation in the risk of chronic NCDs, participants were recruited from households, primary healthcare units and fitness centres located within the defined geographical areas.

Once their eligibility was confirmed, participants were explained the study protocol and invited to take part. After obtaining informed written consent, participants were questioned on their demographic background and health. Participants were identified as being ‘at risk of chronic NCD’ by the presence of two or more metabolic risk factors (hypertension, dyslipidaemia, diabetes, obesity) based on their past medical history, documental evidence based on clinic records, drug prescriptions, laboratory testing and anthropometry. Those without any risk factors were considered as apparently healthy adults. Further, those who met three or more PA targets were classified as ‘high active’ and others as ‘low active’ [26], based on the technology-enabled personalised feedback obtained in the main study [24].

To explore barriers and facilitators of being active, face-to-face semi-structured in-depth interviews were conducted for approximately 20–25 minutes with each participant in the privacy of their home, fitness centre or clinic in a quiet place with minimum disturbance. Repeat interviews were not carried out and field notes were not made during the interviews. The interviewer (MP) who had graduated from a reputed state university in Sri Lanka with a Bachelor of Science (Special) degree in physical education, was employed as a research assistant to the project at the time of data collection. He had experience in qualitative interview techniques and was further trained by a team of experts including, a consultant community physician, two exercise physiologists and a health psychologist. The interviewer did not have any professional or personal relationship with the participants prior to the interviews and any prior assumptions or bias towards the research topic. Participants were informed that the research intended to explore their natural thoughts and ideas and that there were no right or wrong answers to the questions asked, and therefore to be honest and provide impartial responses.

Interviews were guided using a pre-tested interview topic guide, which was based on a previous study [27] and adapted to the local setting [24]. It included four broad open-ended questions and 1–5 prompts for each question to gauge the participants’ understanding of PA, clarify what type of PA they do, what helps them to be active, reasons for taking part in PA, reasons for not doing PA, perceptions of their level of PA compared to others, family or peer attitudes towards PA and how family/peers influence their PA. The final set of questions were about expected benefits from PA, and motives and barriers to PA.

Each interview was recorded using a digital audio-recorder and transcribed in the local language, before being translated into English for analysis. Debriefing sessions with other investigators were conducted after the first few interviews to discuss the progress of interviews. These involved; discussion of any questions that were not generating detailed answers, participant responses that may indicate relevance, or areas useful to routinely seek further clarifications, and the research assistant’s overall perspective on the depth of insight gained. No issues were identified, as the topic guide generated detailed and engaging feedback, so it remained unchanged. We attempted to contact participants following interviews to seek their feedback on their transcripts, but most were unreachable, and those who we spoke to were unwilling to contribute this additional time.

All the transcriptions were anonymised (removing names and addresses) to secure confidentiality of the respondents.

### Data analysis

The transcripts were analysed using framework analysis, which involves transcription, familiarization with data, coding, constructing an analytical framework and developing a framework matrix [28]. The transcripts were first read by the interviewer (MP) to ensure familiarity with the contents prior to being translated into English by another independent translator. Next, a sample of transcripts were coded independently by three members of the research team (MP, FG, CA) and considered alongside themes derived in previous similar studies [19, 20, 29–33], to form a comprehensive framework of a-priori themes and sub-themes in which to classify participant responses. Thereafter, the remaining transcripts were coded deductively by one researcher (MP) according to the identified themes, and the distribution and clarification of sub-themes was then refined iteratively in collaboration with FG.

## Results

Seventy-seven adults were recruited for the main study. Of them, 35 were approached for consent and interviews for the current study, while attempting to reflect as much as possible the variation of the target population. Four subjects declined to participate in interviews. After interviewing 28 participants, the data saturation point was reached. To verify that the data saturation was achieved, three more participants were interviewed with no new ideas emerging, increasing the total number of interviewed participants to 31 for the current study. The interviewed participants included 20 apparently healthy adults (mean age 39 years; age range 21–58) and 11 adults at risk of chronic NCDs (mean age 45 years; age range 31–52). All belonged to ‘low active’ category based on the technology-enabled personalised feedback. Other basic characteristics of the participants are summarised in Table 1.

**Table 1:** Socio-demographic and anthropometric characteristics of the participants.

Characteristics	Participants (%)
**Sex**	
Male	15 (48.4%)
Female	16 (51.6%)
**Age (years)**	
20–39	12 (38.7%)
40–59	19 (61.3%)
**Ethnicity**	
Sinhalese	28 (90.3%)
Others	3 (9.7%)
**Health status**	
Adults at risk of NCD	11 (35.5%)
Apparently healthy adults	20 (64.5%)
**Current marital status**	
Single	5 (16.1%)
Married	26 (83.9%)
**Highest level of education**	
Grade 6–10	5 (16.1%)
Passed G.C.E. (O/Level) *	5 (16.1%)
Passed G.C.E. (A/Level) *	6 (19.4%)
Vocational training	5 (16.1%)
University education	6 (19.4%)
Post-graduate education	4 (12.9%)
Monthly family income (USD)*	
> 300	17 (54.8%)
201 to 300	4 (12.9%)
101 to 200	9 (29%)
≤ 100	1 (3.2%)
**Current employment status**	
Employed (Full-time)	25 (80.6%)
Employed (Part-time)	1 (3.2%)
Self-employed	3 (9.7%)
Unemployed	2 (6.5%)
**Current smoking status**	
Smokers	5 (16.1%)
Non-smokers	26 (83.9%)
**Body composition (based on BMI)**	
Normal	8 (25.8%)
Overweight	12 (38.7%)
Obese	11 (35.5%)

*GCE = General Certificate of Education; O/L = Ordinary Level; A/L = Advanced Level; USD = United States Dollar; NCD = Non-Communicable Diseases

### Theme 1: Perceived barriers to physical activity

All the participants reported at least a few barriers for being active, which are grouped into the 12 clusters as set out in Table 2.

**Table 2:** Sub-themes on barriers for physical activity.

Sub-themes	Example of quotes
Time	*“Physical activities are needed but time is a challenge*. *One of the reasons for not engaging in activities is that I don’t have enough time”*. *(Female*, *NCD risk*, *<40)*
	*“Honestly*, *I do not have time to be involved in regular exercise for health benefits*. *I can’t manage my time*, *and I am too busy”*. *(Male*, *healthy*, *<50)*
Long working hours	*“We don’t have a specific time to work*, *as in we go to work at 5 am and come home around 11 pm or 1 am in the next morning or even sometimes*, *we come home at 6 am and go back to work at 7 am on the same day*. *So*, *no time to exercise at all”*. *(Male*, *healthy*, *<30)*
	*“The only problem*, *according to my job is that I frequently do night shifts*, *I am actually doing 24-hour shifts*. *I am working about 4 days a week*, *also not getting three full days off*. *I can be free on average only for 48 hours”*. *(Male*, *healthy*, *<40)*
Family commitments	*“Obstacles for me to be active*.* *.* *. *is my two babies*. *They are always on my mind*, *otherwise no obstacle at all”*. *(Female*, *healthy*, *<40)*
	*“Actually*, *in the past*, *when my children were studying*, *when they go to tuition classes*, *I have to cook for them in the morning*. *Then my husband goes on walks alone*, *I am not going because I want to make food for them*. *So next*, *even on weekdays*, *most of the time my husband walks around the house alone*, *I don’t get a chance to walk like that because I have to cook in the morning before I go to school at 6*.*30 in the morning”*. *(Female*, *NCD risk*, *<60)*
Physical resources	*“We don’t have facilities to do exercise at home; we don’t have exercise equipment at home*, *so we are unable to do exercise when we have free time in our busy lives”*. *(Male*, *healthy*, *<60)*
	*“Not enough resources are also a problem*. *If there is a swimming pool at home or nearby*, *you can easily go for a swim*. *When you have to go to a place far from your home*, *you feel lazy and get demotivated”*. *(Female*, *healthy*, *<50)*
Safety	*“Sometimes*, *we feel insecure about the environment*. *If there are street lights and CCTV camera*, *we feel secure*. *The walking pathway in [place] is well secured; policemen are always on the watch*. *Since that area is small it is easier to get help unlike in larger areas*. *[place] is too large and [place] is open to the main road”*. *(Female*, *NCD risk*, *<50)*
	*“Without kids I try to walk but with them it’s difficult and unsafe*. *They let go of my hands and run*. *Even for small distances like going to the pharmacy*, *I use a vehicle because it is really unsafe to walk in these roads with my kids”*. *(Female*, *NCD risk*, *<40)*
Weather	*“As an example*, *due to heavy rain*, *we had a break in our walking routine and gradually the entire thing stopped”*. *(Female*, *NCD risk*, *<50)*
	*“We are unable to go [for exercise] if it rains*, *otherwise we can go*. *If it rains in the morning we stay inside*. *It is very difficult for us to exercise outdoors in rainy weather*. *It feels lazy to go for a walk on a rainy day”*. *(Male*, *healthy*, *<50)*
Discomfort	*“I easily become tired*. *I have wheeze (pause)*. *When I walk to my mother’s or sister’s place*, *I feel difficult to talk for about 5 to 10 minutes because of wheezing*. *Once I bought an exercise machine*, *but I couldn’t do exercise at least for 5 minutes because I felt extremely tired whenever I used it; so*, *I have put that away and might end up in a more severe situation”*. *(Female*, *NCD risk*, *<40)*
	*“Probably due to my obesity*, *I feel tired and fatigue quickly when I exercise*. *Sometimes I have difficulty in breathing when I exercise”*. *(Male*, *NCD risk*, *<40)*
No priority for doing physical activity	*“From the moment we get up in the morning*, *all we do is attending to our day-to-day needs*, *which is more important for us*. *So that we are not left with any time to spare for our physical wellbeing”*. *(Male*, *healthy*, *<60)*
	*“Most likely I feel*, *it is because we do not consider it [exercise] as part of life*. *If we think that we need exercises as much as what we eat and drink*, *time will automatically get allocated for exercises*. *May be we have not been thinking like that”*. *(Female*, *healthy*, *<50)*
Lack of understanding of the importance of physical activity	*“We don’t think about exercise much*, *instead we control diet*. *We only control our diet*. *We concern about our diet more than exercise”*. *(Female*, *healthy*, *<40)*
	*“I don’t know anyone among us who exercise for preventing diseases*. *Since we all are young people*, *no one among us think about preventing diseases through exercise*. *So*, *I think it is not essential for young people like us”*. *(Male*, *healthy*, *<40)*
	*“My BMI is anyway low (laughs)*, *I am thin*. *So*, *no one tells me to exercise*, *because I am thin*. *Thus*, *no one force me or advise me to exercise”*. *(Female*, *healthy*, *<30)*
Not having an exercise partner	*“One reason is that our friends have left from this neighbourhood for jobs or after marrying*. *So*, *I have no companions anymore… After my cousin left abroad*, *there is a void*. *Also*, *since wife has the baby*, *she too has commitments”*. *(Male*, *healthy*, *<40)*
	*“The problem is that most of the evenings there is no one to walk with*. *If my husband is at home*, *we both go for a walk*. *If I want to go out for a walk*, *I have to travel a bit far from home to a walking track*. *So*, *that’s the problem; I can’t travel there alone*. *So*, *going for a walk without a partner is a problem”*. *(Female*, *NCD risk*, *<60)*
Family is not supportive of physical activity	*“Actually*, *I don’t receive any support from the family to do exercise*. *I do all the domestic chores alone*. *So*, *I don’t have much time to exercise”*. *(Female*, *healthy*, *<40)*
	*“My parents don’t have much knowledge about it [exercise]*. *Sometimes they scold me by saying things like why are you exerting yourself so much*. *Usually*, *family and friends don’t know about exercise*. *Even friends say that going to the gym is useless and it’s a waste of time; instead*, *you can have a nice sleep at home”*. *(Male*, *healthy*, *<30)*
Physical limitations	*“Now I feel I am very weak than in the past*. *Although if I start to exercise*, *sometimes I might not be able to do it properly*. *I have such a doubt as well*. *I feel tired now*, *and I am weak*.* *.* *. *I think even if I start to exercise I*, *would not be able to continue it”*. *(Female*, *healthy*, *<50)*
	*“Most of the time*, *I am walking*. *I am not running because of my body weight; it is too high; so*, *it might cause back pains if I run a long distance”*. *(Male*, *NCD risk*, *<50)*
	*“I used to go to a gym before I hurt my knee*. *However*, *now I am not participating in any strenuous exercise because my knee cartilage is partially damaged”*. *(Female*, *NCD risk*, *<60)*

Time was a common barrier identified by the sample. Time constraints were linked to both males and females working long hours, long commutes and having commitments such as housework and childcare. Interestingly, a few young apparently healthy adults had misconceptions about PA, such as the superiority of dietary control over exercise for maintaining health and not requiring exercise if having a lower body mass index. In contrast, the majority of adults at risk of chronic NCDs considered PA a high priority and reported good understanding of its importance. For some, the barriers were related to having negative experiences of doing exercise. For example, particularly those who were overweight or had obesity and those at risk of chronic NCDs found exercise to be unpleasant because they did not feel fit enough, got tired easily or experienced pain. A few participants reported feeling a lack of social support, whether in the form of having someone to exercise with or lack of encouragement from family members. A few barriers were also attributed to the physical environment such as weather and safety on the road.

### Theme 2: Facilitators of physical activity

All the participants reported at least one motive for engaging in PA or exercise (Table 3).

**Table 3:** Sub-themes on perceived facilitators of physical activity.

Sub-themes	Example of quotes
Maintain or improve health	*“About the factors I got motivated to do exercises*, *I decided to do exercise after my cholesterol went up*.* *.* *. *Actually*, *I initiated exercise to burn fat*. *That is my motivating factor”*. *(Male*, *NCD risk*, *<40)*
	*“One thing is that physical activity makes us healthy*, *and also it prevents diseases and aging*. *Some don’t believe that I am 36*. *Because when I meet some of my own age persons*, *they are so fat*, *with grey hair; I am also getting a few now… So*, *I have such benefits”*. *(Male*, *healthy*, *<40)*
	*“Sports are good for the mental wellbeing too*. *Then*, *when any family problem is encountered*, *when playing sports*, *it tends to forget everything*. *It balances off…” (Female*, *healthy*, *<40)*
Improve fitness and reduce lethargy	*“Another reason for me to do physical activity is to improve strength*. *That means when we do certain activities*, *we realise that our muscles are not strong enough*. *Therefore*, *I do exercise to strengthen my muscles”*. *(Male*, *healthy*, *<60)*
	*“I’m lethargic when I stay home but when I engage in physical activities I feel more fresh and active*. *Also reduces the lethargic feeling”*. *(Male*, *healthy*, *<30)*
Enjoying physical activity or like to do sports	*“I like to do swimming and I enjoy it*. *I used to do a lot of swimming with my friends in the past*. *Now I do it with my child*. *I play badminton with my child also*. *I was in the school badminton team back in the day*. *… Normally*, *when you do a sport you feel joy”*. *(Female*, *healthy*, *<50)*
	*“Whenever I feel uncomfortable with my body*, *I do physical activities*. *I really enjoy these activities and I motivate myself to do it*. *Sometimes I think how to manage my time*. *Somehow*, *I make up my mind to do these exercises even for an hour”*. *(Female*, *NCD risk*, *<60)*
Habit or custom to exercise	*“One is due to genetics*, *that is my father has done a lot of sports; also*, *all in that generation have done sports and have the genetics*. *So*, *I too since 12 years of age*, *I have been doing exercises”*. *(Male*, *healthy*, *<50)*
	*“My mother*, *sister and sister’s daughter have participated in a recent marathon event organized by the ministry*. *My son and sister’s son intended to participate*, *but they couldn’t because they went on a trip*. *Otherwise*, *all of our family has participated in that marathon event*. *My mother won second place*, *and my sister won 15th place at last year’s event*. *All of our family is inherently sporty (laughs)*. *So*, *I also have that habit of doing sports and exercise regularly”*. *(Female*, *healthy*, *<40)*
Social factors	*“Yes*, *well if they invite me even though I don’t want to I go… Sometimes my friends’ children ask me ‘aunty shall we go*, *mother is asking you to come*.*’ I still go even I feel like it is a nuisance*. *But because of them also it affects me in a good way*. *That’s good for our health also”*. *(Female*, *NCD risk*, *<50)*
	*“If I compare myself with others*, *I think my level of activity is not sufficient*. *So*, *now I know I am not active enough*, *that’s why I put this much of effort for doing exercise”*. *(Male*, *NCD risk*, *<40)*
	*“Well*, *my main intended benefit of exercising is that we can get together as a family*. *So*, *I am more concerned about mental and social wellbeing while doing these [walking] for my physical well-being*. *When the family gets together there’s mental and social happiness”*. *(Female*, *NCD risk*, *<50)*
Being told by others to do physical activity	*“The doctor told me that he can’t give me medicine as I am not yet old for it; and asked me to reduce it through diet and exercises”*. *(Male*, *NCD risk*, *<40)*
	*“Back in the day I was told at least to run to reduce the size of my belly*, *but actually I didn’t do that”*. *(Male*, *healthy*, *<30)*
	*“You earlier asked about others persuading me to do exercises*.* *.* *., *only now I remembered that I went to watch a bodybuilding meet last Saturday*, *and I was told that I must participate next year*. *So*, *that was motivating”*. *(Male*, *healthy*, *<30)*
Physical resources	*“There is a walking track nearly one kilometre away from my house*. *I don’t have any disturbance there*, *and I can freely do my exercise*. *So*, *having such facilities nearby is motivating”*. *(Male*, *NCD risk*, *<50)*
	*“I think there is no shortage of resources*. *Being active does not mean going to a gym*. *We can walk*, *there is a ground*, *and there is a jogging track*. *The jogging track is close to our house*. *Here you can see it [pointing outside the window]*, *our jogging track in [place]”*. *(Male*, *healthy*, *<40)*
Functions	*“Those physical activities are something you have to do daily*, *may be as a job or as a part of my occupation*. *Additionally*, *I have to somehow do those house hold work also”*. *(Male*, *healthy*, *<50)*
	*“At the time of training*, *most of the times I play with school athletes in the ground*. *I play with them and also judge them while training them*. *So*, *that’s a good opportunity for me as well”*. *(Female*, *healthy*, *<40)*

Participants commonly believed that PA would help to prevent diseases, maintain health, and increase their lifespan. In addition, some participants mentioned cosmetic reasons such as to maintain ideal body weight/shape and prevent aging effects were considered for being active. Some participants were motivated by the benefits of physical fitness, such as feeling fresh and being less lethargic. The long-term physiological changes and adaptations to exercise, such as improving brain function, breathing and blood circulation were also recognised by some. Some participants seemed to enjoy PA, such as ‘feeling good’ noted among those at risk of NCDs. Although participants in the sample considered disease prevention as an important motive, it was acknowledged to a lesser extent by adults at risk of chronic NCDs who were concerned about reducing their symptoms as a result of PA.

With regards to psycho-social benefits, PA was perceived as a means of relieving stress and building resilience, while some even used PA for protecting the unity of the family as it was considered an opportunity to get together as a family and thereby nurture intra-family interactions. Participants found positive attitudes of family members, having peers who exercise and seeing their peers being more active than themselves to be motivating. Similarly, advice they received from doctors or family was also considered motivating for the majority who received it. Albeit to a lesser extent than health or social reasons, access to exercise equipment, facilities or resources in the office, home or in a community setting, were reported to encourage active behaviour.

## Discussion

The aim of this study was to better understand the barriers and facilitators of PA among urban dwelling adults in Sri Lanka. In Sri Lanka, local built environments have been modified since 2012, with the development and rejuvenation of urban parks and trails (especially in the Capital of Sri Lanka, Sri Jayewardenepura Kotte) and to facilitate PA of urban dwellers [15, 16]. However, the actual use of these facilities is limited compared to other countries [34], suggesting additional barriers and motives influence participation in PA even in this more PA-friendly environment.

### Barriers to physical activity

Twelve sub-themes were needed to catalogue the many barriers that participants reported to being active. These included competing priorities (e.g., work, family), physical concerns (e.g., local safety, weather), health concerns (discomfort, physical limitations), resources (e.g., local facilities, social support) as well as lack of understanding of the importance of PA for health.

Lack of time was a frequently reported barrier for PA. This finding was compatible with another study from Sri Lanka in a semi-urban city [35] and studies among other South Asians living in India [20], Nepal [21] and the UK [19, 29]. Lack of time has also been cited as a barrier in developed countries like Australia [36], UK [8], Netherlands [37] and USA [38]. This is probably due to demanding jobs in competitive environments across the globe, including in South Asia [21, 22, 29] and as noted in our study. In addition to this, our findings confirm that child caring and family commitments are also considered top priorities which limit the time available for recreational PA. This highlights a common barrier in the Asian culture for considering exercise a low priority [20, 21, 30], unless the benefits following exercise are noticeably advantageous for family care [29, 30]. On the other hand, some argue that lack of time is merely an excuse for not being able to prioritise [31]. In concurrence, most participants in our sample who highlighted time as a barrier, later acknowledged that they were actually less interested in allocating a separate time for exercise in their routine or preferred resting to exercise.

Previous studies across other countries suggests that people report lack of exercise due to feeling ‘too lazy’ after a day’s work or household chores [35] and counting such chores as physically productive [20, 39]. In addition, having beliefs that healthy eating is an effective substitute for PA [40, 41], that PA is not enjoyable [8], and that only overweight or people with chronic diseases should prioritise exercise [20] play a significant role. These misconceptions are mostly reported from lower middle-income countries, and are dependent on health status of the people, as much as on their level of education, gender and age, as shown in other studies [42].

In contrast to previous findings in South Asia, some adults in our sample identified the health impact of their household chores to be trivial, as they claimed that many of these activities are performed slowly or not done manually. Yet, many made no additional attempts to be active, owing to their perception that they are relatively active compared to their close associates (family, friends & neighbours). This perception which could be due to being uncertain on how much PA can be beneficial to health, could affect one’s intentions to be more active. Such vague knowledge about PA guidelines and goals has been evident in Sri Lankan adults according to previous studies [22, 43].

According to previous studies, engagement in physical activity among people with diabetes and arthritis [20, 22, 31, 44] is undermined by uncertainty about what type and level of PA is appropriate and safe for their condition [30, 43] as well as fear of failure often accompanied by low self-efficacy [45]. In concurrence, our sample too showed fear of failure and low self-efficacy in participants carrying out recreational exercise, which is unfamiliar to them. This highlights that in addition to providing PA-friendly environments, building self-efficacy is important for those unaccustomed to perform structured exercise.

Only a few healthy adults and adults at risk of chronic NCDs in our sample reported environment related barriers, such as ‘bad weather’ as a restriction to being active. In a tropical country like Sri Lanka, rain fall throughout the year as much as high temperature could be strong contributors, in contrast to seasonal changes identified as a barrier to recreational exercise in cold climate countries [32]. Only heavy rain fall was cited as a barrier among those who reported environmental barriers in our study, highlighting that the PA habits of most participants were not influenced by the tropical climate. In concurrence, the average rainfall has been the most important factor of concern for using urban parks compared to other weather/climate indicators such as temperature [34]. As for access to facilities, only a few participants identified it as a barrier, highlighting the PA-friendly environment as a strength in urban cities in Sri Lanka. However, the accessibility and usability of PA facilities in the study area could be further enhanced by monitoring automobile traffic and ascertaining the sense of safety in the area.

### Facilitators of taking part in physical activity

Participants were able to recognise many positive reasons to exercise, including health benefits and enjoyment, as well as factors that helped them to maintain their involvement, such as encouragement from others and embedding physical activity as part of daily routine.

Past work has found clinical populations to be motived by health benefits, for example among patients with diabetes in Nepal [21], who were inspired by the feeling of symptom alleviation and other benefits, such as improvement in physical fitness or ability to maintain ideal body shape as a result of PA participation. Our study supports this, finding that adults at risk of chronic NCDs typically showed a positive attitude towards integrating PA into their daily life as they were aware of its importance for their health. Our participants also reported enjoyment, relaxation and satisfaction brought about by exercise, which has not previously been reported in South Asian cultures.

In our participants with and without existing health risk, motivation was attributed to having social support from family, peers and health professionals. This finding is compatible with previous studies conducted in different cultures [21, 32, 41, 46], highlighting the role of social support as a universal facilitator. The need for social support appears to be greater among South Asian women, whose behaviour is largely determined by societal approval and cultural acceptability [19–21, 39]. However, this predefined role of women as homemakers confined to the house [20] was not apparent in our sample, as in most cases the families of the women we interviewed did not oppose exercising even in fitness centres, and who contributed to household chores and child-care. This positive societal approval is further supported by previous studies showing gender equity of using recreational parks in Colombo [34] and young females not being ashamed of exercising in public places [35]. Yet, competitive sports and muscle strengthening exercises seemed less acceptable for being feminine. Further, similar to in Nepal [21], most adults at risk of chronic NCDs in our sample found medical advice to be motivating. Continuous social support from valued others has the potential to foster a sense of ‘relatedness’ to others, which is a factor predicting the establishment of autonomous motivation for physical activity [33].

### Strengths and limitations

This is one of the first studies to report on the barriers and facilitators of PA perceived by urban dwellers in PA-friendly environments in Sri Lanka. The sample included younger and older adult men and women of varied health status and demonstrated considerable consistency among the points raised. Although the main study [24] was a pilot study, the current study was a full-scale qualitative study, and we determined its sample size by achieving the point of data saturation for the current study. Given the lack of research into PA in tropical countries, these findings provide valuable confirmation of how and in what ways there are consistencies or differences between people living in these countries and the non-tropical countries on which most research is based. While we drew some preliminary comparisons between participants with or without the risk of chronic NCDs, the study design and participant numbers did not permit firmer definitive comparisons so these are suggested as potential directions for further study. As the study was based in one urban area of Sri Lanka, we were not able to capture the views of people of all ethnicities present in the population, or the impact of rural settings on working conditions, resources and opportunities to be active. Similarly, as a large proportion of our sample were overweight, our findings may not represent the views of the wider healthy-weight population. The credibility of our study could have been enriched if results were shared with participants and all the interview transcripts were double coded independently with a double assignment independently to a-priori themes was performed.

## Conclusions

As in other parts of the world, time is a common barrier for being physically active among urban dwellers in Sri Lanka who live in PA-friendly environments with urban parks and trails. Tropical weather and facilities were rarely identified as barriers. Although study participants considered that household chores alone were not sufficient to fulfil their daily PA requirement for health, they rarely made concerted attempts to be more active, mainly because they preferred or felt required to give priority to family and social responsibilities over their health. However, when they did take part, people of all ages, and those with disease risk factors including overweight and obesity often reported enjoying and valuing PA. Health benefits and social support were identified as other major facilitators towards being active. Unlike in other South Asian cultures, females too appeared to have enough freedom on their choice for being physically active. The findings suggest that improving physical environments alone is not sufficient to increase PA levels. Greater support to change working time demands and shift attitudes may also be needed.

## References

[1] Booth FW, Gordon SE, Carlson CJ, Hamilton MT. Waging war on modern chronic diseases: primary prevention through exercise biology. J Appl Physiol. 2000; 88(2): 774–787. doi: 10.1152/jappl.2000.88.2.774 10658050

[2] Bull FC, Bauman AE. Physical inactivity: the “Cinderella” risk factor for noncommunicable disease prevention. J Health Commun. 2011; 16 Suppl 2: 13–26. doi: 10.1080/10810730.2011.601226 21916710

[3] Ding D, Lawson KD, Kolbe-Alexander TL, Finkelstein EA, Katzmarzyk PT, van Mechelen W, et al. The economic burden of physical inactivity: a global analysis of major non-communicable diseases. Lancet. 2016; 388 (10051): 1311–1324. doi: 10.1016/S0140-6736(16)30383-X 27475266

[4] Guthold R, Stevens GA, Riley LM, Bull FC. Worldwide trends in insufficient physical activity from 2001 to 2016: a pooled analysis of 358 population-based surveys with 1.9 million participants. Lancet Glob Health. 2018; 6(10): e1077–e1086. doi: 10.1016/S2214-109X(18)30357-7 30193830

[5] Bull FC, Al-Ansari SS, Biddle S, Borodulin K, Buman MP, Cardon G, et al. World Health Organization 2020 guidelines on physical activity and sedentary behaviour. Br J Sports Med. 2020; 54(24): 1451–1462. doi: 10.1136/bjsports-2020-102955 33239350 PMC7719906

[6] Bauman AE, Reis RS, Sallis JF, Wells JC, Loos RJF, Martin BW, et al. Correlates of physical activity: why are some people physically active and others not? Lancet. 2012; 380(9838): 258–271. doi: 10.1016/S0140-6736(12)60735-1 22818938

[7] Rhodes RE, McEwan D, Rebar AL. Theories of physical activity behaviour change: a history and synthesis of approaches. Psychol Sport Exerc. 2019; 42: 100–109.

[8] Withall J, Jago R, Fox KR. Why some do but most don’t. Barriers and enablers to engaging low-income groups in physical activity programmes: a mixed methods study. BMC Public Health. 2011; 11: 507. doi: 10.1186/1471-2458-11-507 21711514 PMC3141466

[9] Atkinson NL, Permuth-Levine R. Benefits, barriers, and cues to action of yoga practice: a focus group approach. Am J Health Behav. 2009; 33(1): 3–14. doi: 10.5993/ajhb.33.1.1 18844516

[10] Hosseini H, Moradi R, Kazemi A, Shahshahani MS. Determinants of physical activity in middle-aged woman in Isfahan using the health belief model. J Educ Health Promot. 2017; 6: 26. doi: 10.4103/jehp.jehp_68_15 28584826 PMC5441191

[11] Ng SW, Norton EC, Popkin BM. Why have physical activity levels declined among Chinese adults? Findings from the 1991–2006 China Health and Nutrition Surveys. Soc Sci Med. 2009; 68(7): 1305–1314. doi: 10.1016/j.socscimed.2009.01.035 19232811 PMC2731106

[12] Hills AP, Farpour-Lambert NJ, Byrne NM. Precision medicine and healthy living: The importance of the built environment. Prog Cardiovasc Dis. 2019; 62(1): 34–38. doi: 10.1016/j.pcad.2018.12.013 30639136

[13] Rodriguez DA, Lounsbery MAF, Sallis JF. The Active Living Research 2015 Conference: The Science of Policy Implementation. Environ Behav. 2016; 48(1): 4–12.

[14] Humpel N, Owen N, Leslie E. Environmental factors associated with adults’ participation in physical activity: a review. Am J Prev Med. 2002; 22(3): 188–199. doi: 10.1016/s0749-3797(01)00426-3 11897464

[15] Ratnayake R, Wickramaarachchi N, Wattege P. Urban water body recreational development and revitalizing program in Sri Lanka: public perception and willingness to pay. Bhúmi, The Planning Research Journal. 2017; 5(2): 41–54.

[16] Hettiarachchi M, Morrison TH, McAlpine C. Power, politics and policy in the appropriation of urban wetlands: the critical case of Sri Lanka. J Peasant Stud. 2019; 46(4): 729–746.

[17] Ranasinghe CD, Ranasinghe P, Jayawardena R, Misra A. Physical activity patterns among South-Asian adults: a systematic review. Int J Behav Nutr Phys Act. 2013; 10(1): 116. doi: 10.1186/1479-5868-10-116 24119682 PMC3854453

[18] De Silva Weliange S, Fernando D, Gunatilake J. Pattern of physical activity among Sri Lankan adults in the district of Colombo: a cross-sectional study. Asia Pac J Public Health. 2016; 28(8): 725–736. doi: 10.1177/1010539516660191 27469309

[19] Jepson R, Harris FM, Bowes A, Robertson R, Avan G, Sheikh A. Physical activity in South Asians: an in-depth qualitative study to explore motivations and facilitators. PLoS One. 2012; 7(10): e45333. doi: 10.1371/journal.pone.0045333 23071511 PMC3468573

[20] Mathews E, Lakshmi JK, Ravindran TKS, Pratt M, Thankappan KR. Perceptions of barriers and facilitators in physical activity participation among women in Thiruvananthapuram City, India. Glob Health Promot. 2016; 23(4): 27–36. doi: 10.1177/1757975915573878 25829405 PMC4609289

[21] Kadariya S, Aro AR. Barriers and facilitators to physical activity among urban residents with diabetes in Nepal. PLoS One. 2018; 13(6): e0199329. doi: 10.1371/journal.pone.0199329 29953475 PMC6023206

[22] Medagama A, Galgomuwa M. Lack of infrastructure, social and cultural factors limit physical activity among patients with type 2 diabetes in rural Sri Lanka, a qualitative study. PLoS One. 2018; 13(2): e0192679. doi: 10.1371/journal.pone.0192679 29462186 PMC5819806

[23] Tong A, Sainsbury P, Craig J. Consolidated criteria for reporting qualitative research (COREQ): a 32-item checklist for interviews and focus groups. Int J Qual Health Care. 2007; 19(6): 349–357. doi: 10.1093/intqhc/mzm042 17872937

[24] Arambepola C, Perera M, Gillison F, Peacock O, Thompson D. The understanding, acceptability, and relevance of personalised multidimensional physical activity feedback among urban adults: evidence from a qualitative feasibility study in Sri Lanka. BMC Public Health. 2021; 21(1): 715. doi: 10.1186/s12889-021-10774-0 33849495 PMC8045206

[25] Francis JJ, Johnston M, Robertson C, Glidewell L, Entwistle V, Eccles MP, et al. What is an adequate sample size? Operationalising data saturation for theory-based interview studies. Psychol Health. 2010; 25(10): 1229–1245. doi: 10.1080/08870440903194015 20204937

[26] Western MJ, Thompson D, Peacock OJ, Stathi A. The impact of multidimensional physical activity feedback on healthcare practitioners and patients. BJGP Open. 2019; 3(1): bjgpopen18X101628. doi: 10.3399/bjgpopen18X101628 31049409 PMC6480860

[27] Western MJ, Peacock OJ, Stathi A, Thompson D. The understanding and interpretation of innovative technology-enabled multidimensional physical activity feedback in patients at risk of future chronic disease. PLoS One. 2015; 10(5): e0126156. doi: 10.1371/journal.pone.0126156 25938455 PMC4418766

[28] Gale NK, Heath G, Cameron E, Rashid S, Redwood S. Using the framework method for the analysis of qualitative data in multi-disciplinary health research. BMC Med Res Methodol. 2013; 13: 117. doi: 10.1186/1471-2288-13-117 24047204 PMC3848812

[29] Lawton J, Ahmad N, Hanna L, Douglas M, Hallowell N. “I can’t do any serious exercise”: barriers to physical activity amongst people of Pakistani and Indian origin with Type 2 diabetes. Health Educ Res. 2006; 21(1): 43–54. doi: 10.1093/her/cyh042 15955792

[30] Sriskantharajah J, Kai J. Promoting physical activity among South Asian women with coronary heart disease and diabetes: what might help? Fam Pract. 2007; 24(1): 71–76. doi: 10.1093/fampra/cml066 17179137

[31] Lascar N, Kennedy A, Hancock B, Jenkins D, Andrews RC, Greenfield S, et al. Attitudes and barriers to exercise in adults with type 1 diabetes (T1DM) and how best to address them: a qualitative study. PLoS One. 2014; 9(9): e108019. doi: 10.1371/journal.pone.0108019 25237905 PMC4169586

[32] Korkiakangas EE, Alahuhta MA, Husman PM, Keinänen-Kiukaanniemi S, Taanila AM, Laitinen JH. Motivators and barriers to exercise among adults with a high risk of type 2 diabetes—a qualitative study. Scand J Caring Sci. 2011; 25(1): 62–69. doi: 10.1111/j.1471-6712.2010.00791.x 20384973

[33] Ferrand C, Perrin C, Nasarre S. Motives for regular physical activity in women and men: a qualitative study in French adults with type 2 diabetes, belonging to a patients’ association. Health Soc Care Community. 2008; 16(5): 511–520. doi: 10.1111/j.1365-2524.2008.00773.x 18355245

[34] Pussella PGRNI, Li L. Identification and assessment of the driving forces for the use of urban green parks and their accessibility in Colombo, Sri Lanka, through analytical hierarchical processing. Geospat Health. 2019; 14(1): 72–80. doi: 10.4081/gh.2019.738 31099517

[35] Karunanayake AL, Senaratne CD, Stathi A. A descriptive cross sectional study comparing barriers and determinants of physical activity of Sri Lankan middle aged and older adults. PLoS One. 2020; 15(5): e0232956. doi: 10.1371/journal.pone.0232956 32396547 PMC7217429

[36] Ashton LM, Hutchesson MJ, Rollo ME, Morgan PJ, Thompson DI, Collins CE. Young adult males’ motivators and perceived barriers towards eating healthily and being active: a qualitative study. Int J Behav Nutr Phys Act. 2015; 12(1): 93. doi: 10.1186/s12966-015-0257-6 26169503 PMC4501295

[37] Romeike K, Abidi L, Lechner L, de Vries H, Oenema A. Similarities and differences in underlying beliefs of socio-cognitive factors related to diet and physical activity in lower-educated Dutch, Turkish, and Moroccan adults in the Netherlands: a focus group study. BMC Public Health. 2016; 16(1): 813. doi: 10.1186/s12889-016-3480-4 27534933 PMC4989297

[38] Lee C, Ory MG, Yoon J, Forjuoh SN. Neighbourhood walking among overweight and obese adults: age variations in barriers and motivators. J Community Health. 2013; 38(1): 12–22. doi: 10.1007/s10900-012-9592-6 22811072

[39] Riley L, Mili S, Trinh-Shevrin C, Islam N. Using qualitative methods to understand physical activity and weight management among Bangladeshis in New York city, 2013. Prev Chronic Dis. 2016; 13: E87. doi: 10.5888/pcd13.160077 27390073 PMC4951079

[40] Hu SH, Fu MR, Liu S, Lin YK, Chang WY. CE: Original Research: Physical activity among Chinese American immigrants with prediabetes or type 2 diabetes. Am J Nurs. 2018; 118(2): 24–32. doi: 10.1097/01.NAJ.0000530221.87469.86 29329117 PMC5785420

[41] Alvarado M, Murphy MM, Guell C. Barriers and facilitators to physical activity amongst overweight and obese women in an Afro-Caribbean population: a qualitative study. Int J Behav Nutr Phys Act. 2015; 12: 97. doi: 10.1186/s12966-015-0258-5 26215108 PMC4517402

[42] Metwally AM, Yousof H, Elkholy MM, Eletreby LA, Barakat AA, Abd El Dayem SM, et al. Determinants influencing awareness and healthy practices among a sample of insulin-dependent diabetic Egyptian patients: a rural community-based study. Open Access Maced J Med Sci. 2021; 9(E): 500–508.

[43] Ranasinghe P, Pigera ASAD, Ishara MH, Jayasekara LMDT, Jayawardena R, Katulanda P. Knowledge and perceptions about diet and physical activity among Sri Lankan adults with diabetes mellitus: a qualitative study. BMC Public Health. 2015; 15: 1160. doi: 10.1186/s12889-015-2518-3 26597081 PMC4657222

[44] Metwally AM, Soliman M, Abdelmohsen AM, Kandeel WA, Saber M, Elmosalami DM, et al. Effect of counteracting lifestyle barriers through health education in Egyptian type 2 diabetic patients. Open Access Maced J Med Sci. 2019; 7(17): 2886–2894. doi: 10.3889/oamjms.2019.624 31844454 PMC6901843

[45] Bandura A. Health promotion by social cognitive means. Health Educ Behav. 2004; 31(2): 143–164. doi: 10.1177/1090198104263660 15090118

[46] Mier N, Medina AA, Ory MG. Mexican Americans with type 2 diabetes: perspectives on definitions, motivators, and programs of physical activity. Prev Chronic Dis. 2007; 4(2): A24. 17362615 PMC1893123

